# Subdural Abscess Suspected to Have Developed From Acute Sinusitis via the Foramen Cecum: A Case Report

**DOI:** 10.7759/cureus.77214

**Published:** 2025-01-10

**Authors:** Ryo Maruyama, Masanori Yatomi, Daisuke Yuunaiyama, Haruka Nishimura, Kiyoaki Tsukahara

**Affiliations:** 1 Otolaryngology, Tokyo Medical University, Tokyo, JPN; 2 Otolaryngology - Head and Neck Surgery, Tokyo Medical University, Tokyo, JPN; 3 Radiology, Tokyo Medical University, Tokyo, JPN

**Keywords:** acute sinusitis extra-nasal complication, diplopia, foramen cecum, pediatric acute sinusitis, subdural abscess

## Abstract

The foramen cecum connects the intracranial space to the sinus cavities. However, it is usually blind-ended and typically closed by surrounding bone and tissue. The opening of this anatomical structure can provide a pathway for intracranial infection from acute sinusitis. However, no previous reports have documented the spread of acute sinusitis intracranially via the foramen cecum, to the best of our knowledge. In this case report, we report a case of subdural abscess that was thought to have developed from acute frontal sinusitis through the foramen cecum and provide a review of relevant literature. The patient was a 15-year-old male. He developed right eyelid edema and diplopia after a common cold and was referred to our pediatric department after an MRI revealed sinus and intracranial lesions. Imaging studies suggested the development of a subdural abscess via the foramen cecum. Endoscopic sinus surgery was performed in our department to control the infection, followed by brain abscess drainage in the neurosurgery department. The foramen cecum is an anatomical feature that can connect the inside and outside of the skull, increasing the risk of infections spreading to the brain.

## Introduction

Acute sinusitis is a common condition encountered in pediatric practice, with *Staphylococcus aureus* and *Streptococci* species being the most common causative organisms [1]. Advances in antimicrobial treatments have enabled the successful conservative management of several cases of acute sinusitis. However, it remains a condition with the potential to cause severe complications. Intracranial complications include subdural abscesses, epidural abscesses, meningitis, brain abscesses, and cavernous sinus thrombosis, while intraorbital complications include orbital cellulitis and orbital subperiosteal abscesses [2].

The foramen cecum (FC) is a small bony canal located in the anterior cranial fossa. It lies anterior to the cribriform plate, posterior to the frontal bone, within the frontoethmoidal suture, and anterior to the crista galli [3]. During embryonic development, the FC contains a dural protrusion extending from the anterior cranial fossa to the cutaneous surface of the nose. Normally, this dural protrusion retracts completely, and the FC becomes closed by fibrous tissue lined with periosteum [4,5]. An open FC can serve as a conduit for intracranial infections originating from acute sinusitis [6]. However, our literature search did not yield any reports specifically describing intracranial infections transmitted via the FC.

In this report, we present a suspected case of a subdural abscess resulting from acute sinusitis that likely spread through the FC.

## Case presentation

This is the case of a 15-year-old male. The patient initially presented with a fever, which was suspected to be due to a common cold. This was followed by a nasal discharge that resolved after three days. However, he developed a headache on the 10th day. The fever recurred on the 11th day, reaching 38°C. His consciousness was clear. He developed right eyelid swelling and diplopia in the right eye on the 14th day.

He visited an ophthalmologist, who diagnosed suspected blepharitis and prescribed levofloxacin eye drops and cefditoren pivoxil. On the 15th day, the patient was referred to the ophthalmology department of a general hospital due to persistent eyelid swelling. The examination revealed no significant ophthalmological abnormalities. Ceftazidime hydrate eye drops were added to the treatment, and cefditoren pivoxil was continued.

On the 17th day, the diplopia improved, and intravenous ceftazidime hydrate and oral levofloxacin hydrate were initiated. However, diplopia had reappeared by the nineteenth day, prompting an MRI scan. The MRI revealed sinus and intracranial lesions. Consequently, the patient was referred to the pediatric department of Tokyo Medical University for further evaluation and management.

Initial findings

Upon presentation at the clinic, the patient had a fever of 38.2°C, right eyelid edema, and rigidity of the terminus. Manual muscle testing showed no weakness in the upper limbs; however, lower limb weakness was observed on the right side. Specifically, right thigh flexion and extension were rated 1/5, and right lower leg flexion and extension were also rated 1/5. In contrast, the left thigh had a flexion rating of 5/5 and an extension of 4/5, while the left lower leg had a flexion rating of 5/5 and an extension of 5/5.

Blood biochemistry tests at the initial visit to our hospital showed a white blood cell count of 40,100/mm³, 95% neutrophils, and a C-reactive protein (CRP) level of 26 mg/dL. These findings suggested a bacterial infection. Cerebrospinal fluid (CSF) examination showed a cell count of 311/mm³, with 69.5% polymorphonuclear cells, glucose concentration of 66 mg/dL, and protein concentration of 88 mg/dL. CSF cultures did not identify a causative organism. A diagnosis of bacterial meningitis was based on the clinical and laboratory findings.

Imaging

**Figure 1:**
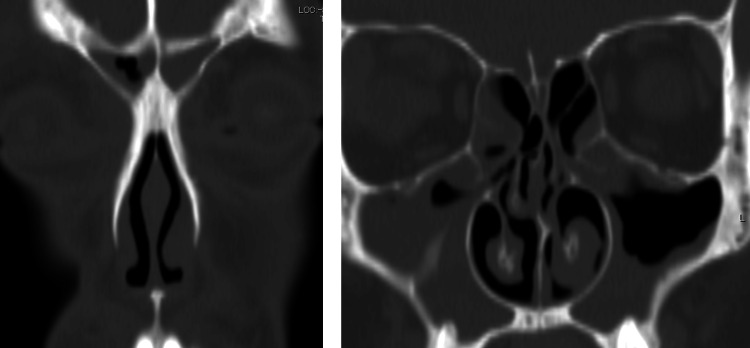
Coronal Sinus CT Coronal section of CT showing soft shadows in the bilateral frontal, maxillary, and ethmoidal sinuses. CT: computed tomography

CT showed soft shadows in the bilateral frontal, maxillary, and ethmoidal sinuses (Figure 1).

**Figure 2:**
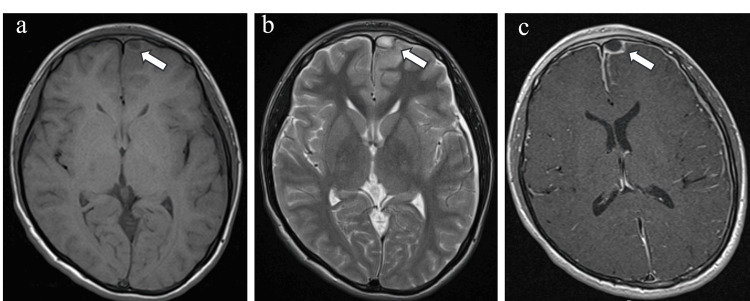
Axial Head MRI a) Horizontal MRI section showing a low signal on T1-weighted images, b) high signal on T2-weighted images in the left frontal epidural space with an approximate dimension of 10×8 mm, c) a uniform low-signal area with contrast effect in the surrounding dura mater on T1-weighted images with gadolinium contrast. A contrast effect was also observed in the frontal bone marrow.

Acute sinusitis was suspected: horizontal MRI section showed a low-signal area of approximately 10×8 mm on T1-weighted images, a high-signal area on T2-weighted images, and a uniform low-signal area with a contrast effect in the surrounding dura mater on T1-weighted gadolinium-enhanced contrast images in the left frontal epidural space. A contrast effect was also observed in the frontal bone marrow (Figure 2).

**Figure 3:**
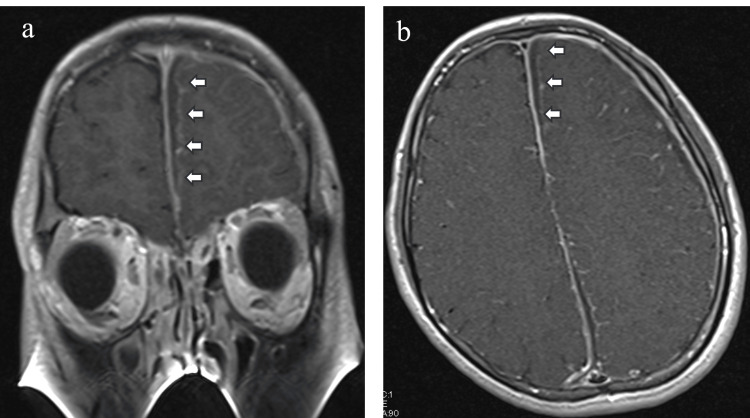
T1-Weighted Gadolinium Contrast Images a) Coronal MRI, b) axial MRI Uniform low-signal areas with contrast effect on T1-weighted gadolinium contrast images were observed under the dura mater along the left interhemispheric fissure.

A low-signal area with a contrast effect was observed in the surrounding dura mater on T1- and T2-weighted images. A uniform low-signal area with a contrast effect in the surrounding dura mater was observed on T1-weighted gadolinium contrast along the left interhemispheric fissure (Figure 3).

**Figure 4:**
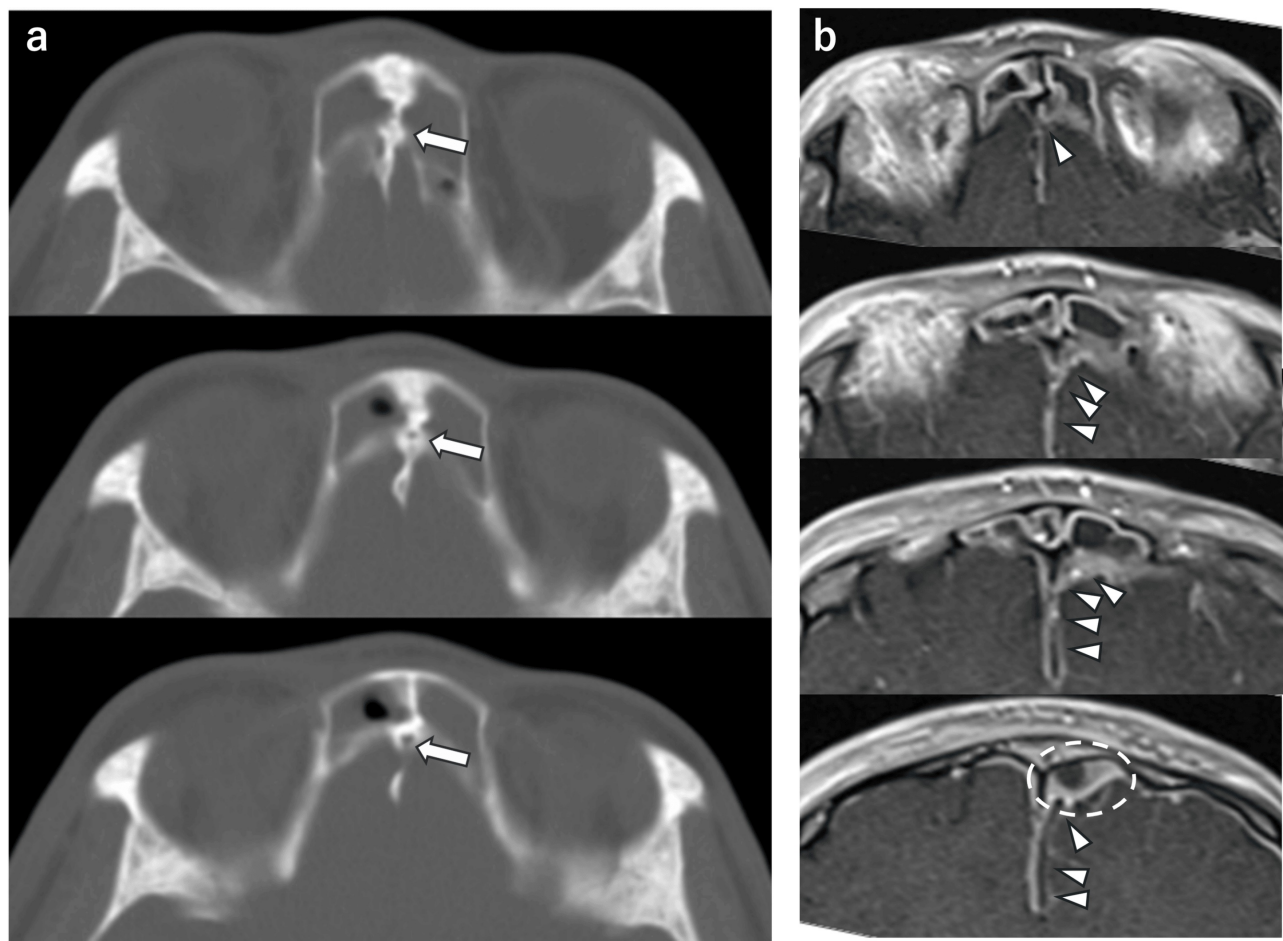
Foramen Cecum a) Axial CT, b) axial MRI T1-weighted gadolinium contrast images. Arrow: foramen cecum; arrowhead: subdural abscess; inside dotted line: subdural abscess CT and MRI T1-weighted gadolinium-enhanced contrast images suggested that an abscess had formed in the epidural region of the left frontal lobe just above the frontal sinus. T1-weighted gadolinium contrast images showed a uniform low-signal area with contrast effect in the epidural region of the left frontal lobe just above the frontal sinus.

A continuous intracranial gap from the right frontal sinus just before the chicken crown was observed on a CT horizontal section (Figure 4).

**Figure 5:**
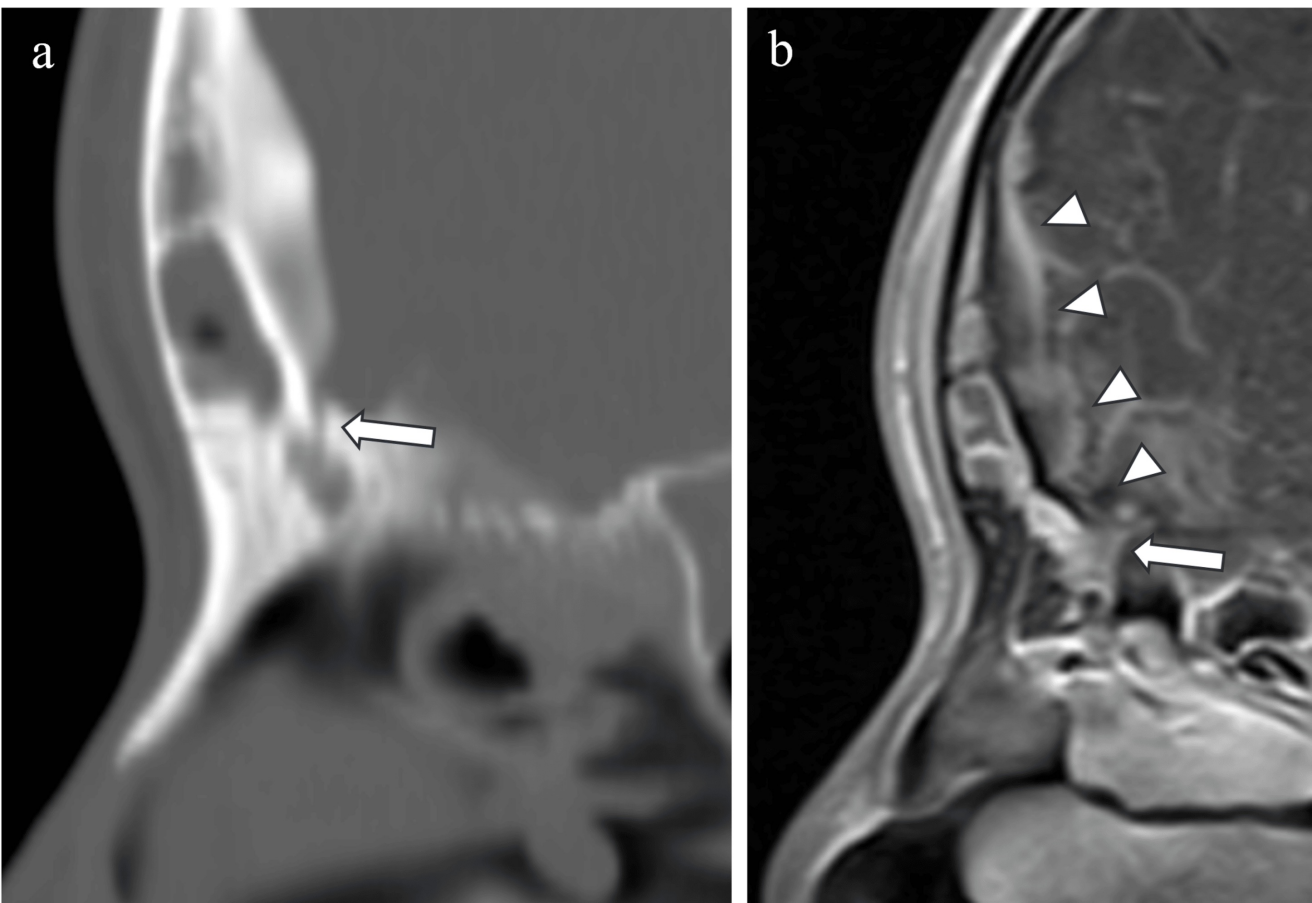
Sagittal Head Figures a) Sagittal CT, b) sagittal MRI The horizontal section shows a fissure from the right frontal sinus to the abscess area. Soft shadows were observed bilaterally in the maxillary, ethmoid, frontal, and sphenoid sinuses.

FC connecting the intracranial and the frontal sinus was seen on the CT sagittal section. A low-signal area was seen on T1-weighted images, and a high-signal area was seen on T2-weighted images. The CT sagittal section showed a continuous gap from the frontal sinus to the intracranial space, and the T1-weighted gadolinium contrast sagittal section of the MR image showed a continuous contrast effect to the epidural abscess at the same site (Figure 5).

Post-hospitalization course

Treatment was initiated with a diagnosis of acute sinusitis, left epidural abscess, left subdural abscess, and bacterial meningitis. Neurosurgical examination revealed no hydrocephalus on imaging, and lumbar puncture findings showed no symptoms of cerebral hypertension. Therefore, the patient was advised to undergo drainage by craniotomy or perforation if the intracranial abscess worsened. The right lower limb paralysis was considered secondary to encephalitis resulting from the epidural and subdural abscesses. The patient was started on meropenem hydrate 6 g three times daily, vancomycin hydrochloride 2.5 g, mannitol 135 g, dexamethasone 6.6 mg, and Keppra 1 g for the treatment of sinusitis, bacterial meningitis, epidural abscess, subdural abscess, and encephalitis. Emergency surgery for infection control was performed in our department on the day after admission.

Intraoperative findings

Bilateral pan-sinusitis was observed, and bilateral endoscopic sinus surgery type IV was performed for infection control. The frontal, ethmoid, maxillary, and sphenoid sinuses were opened and drained on both sides. As much of the inflammatory mucosa as possible was removed from the sinuses. No obvious intracranial communication was found within the right frontal sinus. The nasal and sinus cavities were rinsed to confirm the absence of bleeding, and the surgery was completed. Intraoperative culture showed anaerobic Gram-positive bacilli (*Cutibacterium avidum*).

Postoperative course

Upon admission to the ICU, the patient exhibited MMT2 in the right upper limb and immobility in the right lower limb, with occasional myoclonus-like spasms. The rigidity improved, and fever was absent. Imaging showed no significant changes in the epidural or subdural abscesses (interhemispheric fissure, left frontal lobe, and temporal lobe).

On postoperative day 5, a CSF examination revealed a cell count of 15/mm³ (0% polymorphonuclear cells), glucose concentration of 55 mg/dL, and protein concentration of 51 mg/dL, indicating improvement in meningitis. However, there was no improvement in the right hemiparesis, and intrathecal gentamicin 8 mg was administered, as the subdural abscess had not subsided.

On postoperative day 6, the disorientation worsened. Considering the symptoms indicative of encephalitis, methylprednisolone 1000 mg/day was administered for three days. The right hemiparesis and myoclonus gradually improved following the treatment.

On postoperative day 9, a CSF examination was performed. The results showed a cell count of less than 1/mm³ (0% polymorphonuclear cells), glucose concentration of 102 mg/dL, and protein concentration of 18 mg/dL. Blood tests revealed a white blood cell count of 9,400/mm³, with 85.3% neutrophils and a CRP level of 1.4 mg/dL. This indicated that the inflammation was improving.

**Figure 6:**
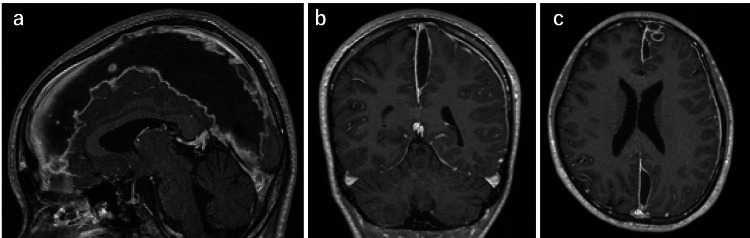
High-resolution 3D T1-weighted turbo field echo imaging of the brain. a) Sagittal, b) coronal, c) axial High-resolution 3D T1-weighted turbo field echo imaging of the brain shows a uniform low-signal area with a contrast effect under the dura along the left interhemispheric fissure. Extensive abscess formation was suspected.

The disorientation improved on postoperative day 13, and the antimicrobial therapy was switched to ceftriaxone sodium hydrate. The patient could perform gross motor activities with the right upper and lower limbs on postoperative day 16. However, MRI angiography showed an enlarged subdural abscess cavity on postoperative day 21, and the patient underwent a craniotomy for drainage by neurosurgery (Figure 6).

Intraoperative findings

An epidural abscess was identified at the anterior margin of the anterior half of the interhemispheric fissure. The left frontal margin was externally punctured, and the abscess capsule was located. The abscess was drained through an incision. A subdural abscess measuring approximately 3 cm in length and 2 cm in width was observed; it was located medial to the midline. The posterior border of the craniotomy was positioned 3 cm anterior to the bregma. The lateral dura was incised 2 cm posteriorly, avoiding damage to the bridging vein and preserving the superior sagittal sinus (SSS). The abscess capsule was identified, incised, and drained.

Intraoperative culture tests failed to identify the causative organism. Examination revealed an interhemispheric fissure and a subdural abscess associated with the left frontal sinus around the frontal cavity. The abscess was drained via craniotomy. The wound was closed after thorough irrigation and drain placement, concluding the surgery.

The patient was discharged from the hospital on postoperative day 48, corresponding to 27 days after the neurosurgery. His disorientation and right hemiplegia had improved significantly. He continued outpatient follow-up for two years, during which no recurrence or residual effects were observed.

## Discussion

A transient dural process is believed to pass through the FC extending from the anterior nasal cavity to the skin of the nasal bridge between the fourth and seventh weeks of gestation [3,7,8]. Following regression of this dural process, a blind edge typically remains at the FC. The FC is often indistinct during neuroimaging [3]. The patency of the FC is common in infants, rare in children, and persists in fewer than 1.5% of adults, where it may remain open without a blind edge [7].

The emissary vein, which connects the nasal cavity to the ventral side of the SSS, may penetrate the FC. This venous anastomosis between the extracranial and intracranial systems is hypothesized to provide a pathway for intracranial venous blood drainage when the SSS is occluded or abnormal [9,10]. Additionally, this venous route has been implicated as a potential pathway for the spread of infection or tumors from the nasal cavity into the intracranial space, as well as a possible entry point for arteriovenous fistulae in the region of the cribriform plate [5,9,11].

FC patency has been considered a factor in intracranial infections, but we found no previous reports directly demonstrating this mechanism on imaging. For the present case, imaging strongly suggested the spread of inflammation from the acute sinusitis into the epidural space via the FC. This pathway is the most likely explanation for the epidural spread observed in this patient.

FCs can present with multiple foramina or a single foramen [12]. For the present case, the FC extended from the intracranial surface into the contralateral frontal sinus. Specifically, the FC opened into the contralateral frontal sinus from within the skull. Inflammatory spillover may result from conditions such as otitis media, trauma, or sinusitis. When frontal sinusitis is accompanied by abscess formation near the frontal lobe, the possibility of inflammatory spillover through the FC should be considered as a potential route of infection.

Antimicrobials for intracranial complications of acute sinusitis should be selected with consideration for resistant bacteria, particularly if the causative organism can be identified [13]. In this case, *C. avidum* was detected in cultures from the sinuses; however, the causative organism was not identified in the spinal fluid or intracranial abscess. This failure to identify the organism may be attributed to prior administration of cefditoren pivoxil, ceftazidime hydrate, and levofloxacin hydrate by a nearby doctor.

For the treatment of meningitis, panipenem-betamipron (PAPM/BP) or meropenem (MEPM) in combination with ceftriaxone or cefotaxime is recommended for immunocompetent patients aged 4 months to <16 years. For immunocompromised patients, a combination of vancomycin and MEPM is recommended [14]. The duration of antibacterial treatment varies depending on the detected pathogen: 7 days for *Haemophilus influenzae* and 10-14 days for *Streptococcus pneumoniae*. For the present case, MEPM and vancomycin were administered for 2 weeks, taking into account the clinical symptoms and the possibility that prior administration of cefditoren pivoxil could have obscured the causative organism.

Surgical treatment is a viable option when intracranial abscesses develop. Some argue that nasal sinus surgery for infection control is not always necessary. However, recurrence of subdural abscess several months after long-term antimicrobial treatment and neurosurgery alone has been reported [15]. For the present case, nasal sinus surgery was performed, antimicrobials were administered, and neurosurgery was considered based on the course of the disease. Sinus surgery was thought to contribute to infection control, as the rigidity of the neck improved after the procedure, and CSF examination showed a decrease in cell count to 1/mm³. Subdural abscesses have a high mortality rate of approximately 10% [16].

## Conclusions

In this case, the general condition and symptoms of the patient gradually improved following sinus surgery and conservative treatment. However, enlargement of the abscess cavity was observed, posing a risk of death. Therefore, surgical drainage should be considered for advanced subdural abscesses to prevent sequelae.
